# Site-specific gene expression analysis using an automated tissue micro-dissection punching system

**DOI:** 10.1038/s41598-017-04616-6

**Published:** 2017-06-28

**Authors:** Takuya Yoda, Masahito Hosokawa, Kiyofumi Takahashi, Chikako Sakanashi, Haruko Takeyama, Hideki Kambara

**Affiliations:** 1 Department of Life Science and Medical Bioscience, Waseda University, 2-2 Wakamatsu-cho, Shinjuku-ku, Tokyo, 162-8480 Japan; 2 Research Organization for Nano &Life Innovation, Waseda University, 513 Waseda-tsurumaki-cho, Shinjuku-ku, Tokyo, 162-0041 Japan; 3 PRESTO, Japan Science and Technology Agency (JST), 5-3 Yonban-cho, Chiyoda-ku, Tokyo, 102–0075 Japan; 4 Computational Bio Big-Data Open Innovation Laboratory, AIST-Waseda University, 3-4-1 Okubo, Shinjuku-ku, Tokyo, 169–0072 Japan

## Abstract

Site-specific gene expression analyses are important for understanding tissue functions. Despite rapid developments in DNA-related technologies, the site-specific analysis of whole genome expression for a tissue remains challenging. Thus, a new tool is required for capturing multiple tissue micro-dissections or single cells while retaining the positional information. Here, we describe the development of such a system, which can pick up micro-dissections by punching a tissue repeatedly in a very short period, e.g., 5 s/sampling cycle. A photo of the punched tissue provides information on the dissected positions, allowing site-specific gene expression analysis. We demonstrate the site-specific analysis of a frozen tissue slice of mouse brain by analyzing many micro-dissections produced from the tissue at a 300-μm pitch. The site-specific analysis provided new insights into the gene expression profiles in a tissue and on tissue functions. The analysis of site-specific whole genome expression may therefore, open new avenues in life science.

## Introduction

A comprehensive understanding of tissue functions requires information regarding cell types as well as site-specific gene expression^1, 2^. For the site-specific gene expression analysis of tissue, imaging technologies, such as *in situ* hybridization, have been a powerful tool^3, 4^. However, only a small number of marker genes can be assessed simultaneously with these methods. For analyzing many gene species simultaneously, the use of a next generation DNA sequencer coupled with an appropriate method for site-specific sampling is required. Furthermore, for understanding tissue functions in detail, site-specific multi-omics (e.g., genomics, transcriptomics, and proteomics) analysis will be necessary. These processes require a sensitive analyzer because the sample sizes are small. Fortunately, several highly sensitive analysis technologies have been developed over recent years. For example, several researchers including our group have reported certain gene expression or genome analysis methods for single cells using next generation DNA sequencers^5–10^. The combination of high throughput analysis of single cells, as well as tissue micro-dissections, with an imaging method provides greater amounts of information on the respective functions than has previously been available. Thus, a method of sampling cells or micro-dissections site-specifically from a tissue should be developed to take advantage of the full potential of analysis technologies.

One candidate is laser capture micro-dissection (LCM). This method enables the isolation of cells or micro-dissections from a tissue slice in a given anatomical area. Specifically, the combination of LCM and various downstream analyses such as RNA-seq and microarray analyses has provided transcriptional landscapes of prenatal^11^ and adult human brains^12^. However, although LCM is a powerful technology, it has several drawbacks, including being limited to use for only fixed and stained tissue, very time-consuming and labor intensive, and not suitable for sampling multiple micro-dissections, the analysis of which is necessary for understanding whole tissue functions. In consequence, as two-dimensional (2D) or three-dimensional (3D) transcriptional maps are useful for comprehending whole tissues, a technology termed serial microtomy sequencing (tomo-seq) has been developed to obtain these maps^12^. With this technique, RNAs are extracted from individual thin tissue cryo-sections or single cells to obtain 2D and 3D transcriptional maps and combine with reference data^13–18^. However, whereas tomo-seq requires multiple replicates of the same samples, the information provided still is associated with a low spatial resolution. Recently, a system employing a capillary tube for manually picking up a single cell from a tissue has been reported^19, 20^, but this requires considerable time to capture up to one hundred cells or more. More recently, Ståhl *et al*. have described a technique for measuring the spatial distribution of transcripts from a tissue slice using bar-coded reverse transcriptase primers immobilized on a glass slide^21^. This is a powerful method for site-specific gene expression analysis but it requires a specific chip for capturing the mRNA. Thus, a high-throughput random access site-specific sampling system for capturing many individual cells or micro-dissections from a tissue remains to be developed.

Based on these considerations, we have reported a system to manually capture a micro-dissection from a plant tissue by using a fine stainless steel hollow needle^22^. In the current study, we have developed a novel system for capturing many micro-dissections automatically in a short period by punching a tissue. In addition to the automated capturing system (punching system), we have also automated our sample preparation technology for single-cell gene expression analysis, using a commercially available dispenser (Caliper-Zephyr (Perkin Elmer)).

Here, we demonstrate that the current system can capture cells from among cultured cells adhered on a plate without damage as well as micro-dissections from a tissue placed in a dish, together with their spatial information. Gene expression in single cells as well as for multiple micro-dissections obtained from a mouse brain slice, using a 300-μm pitch, was analyzed. The insights provided into the tissue gene expression profiles and on tissue functions indicate that the analysis of site-specific whole genome expression using the developed punch technique may have wide-ranging application in bioscience research and potential clinical application.

## Results

### Automated system for capturing tissue micro-dissections as well as single cells

**Figure 1:**
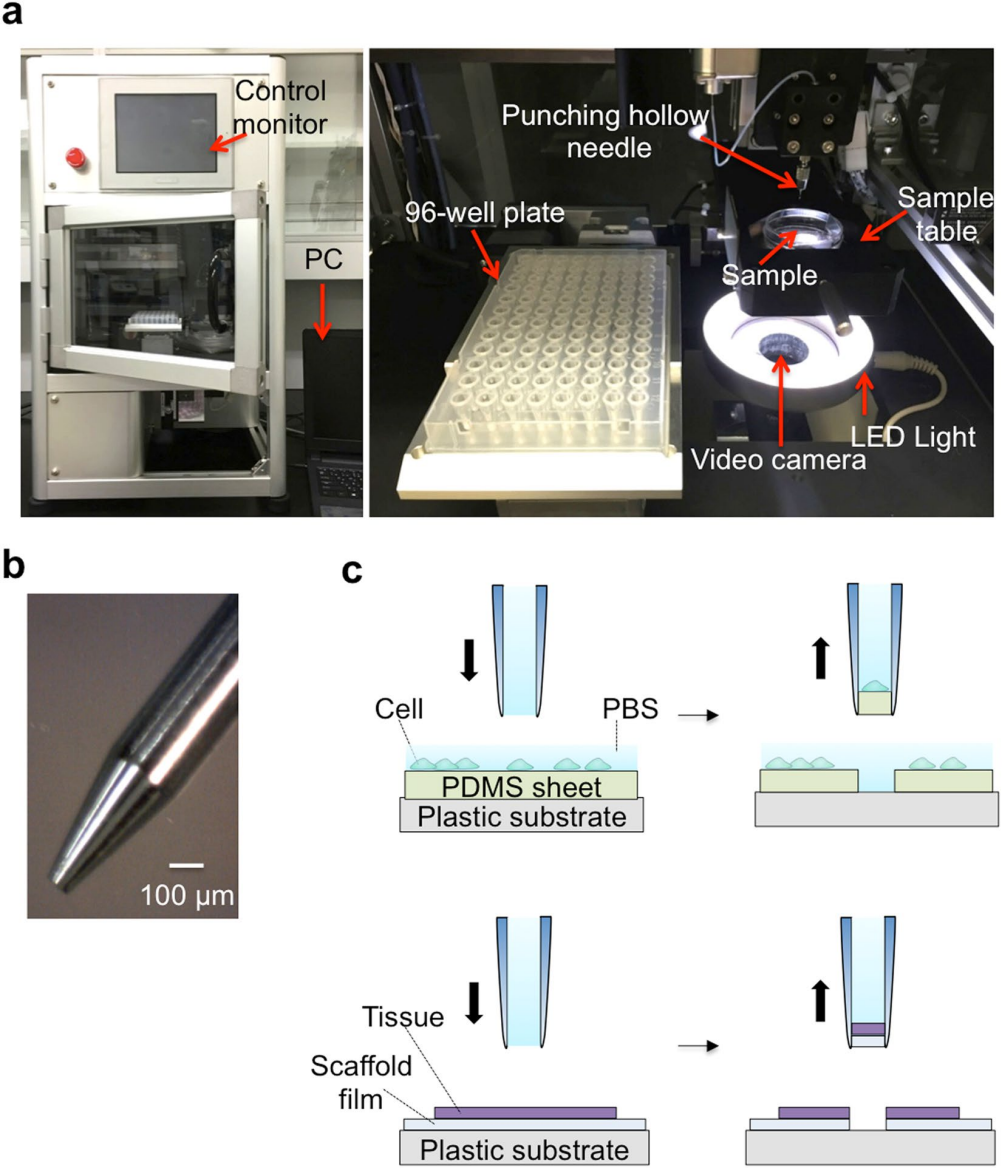
Automatic system for capturing cells and tissue micro-dissections with a hollow punching needle; (**a**) Overview of the capturing system, which consists of a punching unit that is operated by a PC. The sampling points are determined by observing an image of the cells or tissue with a video camera (left). The inside of the system (right). (**b**) Photograph of a hollow punching needle. (**c**) Schematic view of the punching processes for capturing cells (upper) and micro-dissections (lower). A target tissue is placed on a dish coated with a scaffold film. A micro-dissection is produced from the tissue with the film. The sample is held in a hollow punching needle to be carried to the position above a reaction chamber. Then it is ejected into the reaction chamber with a buffer solution.

The capturing system is composed of a punching unit for dissecting a tissue with a hollow punching needle while observing the tissue with a video camera, as well as a control unit and monitor and a PC. An overview of the system and a photograph of the punching unit are shown in Fig. 1a. We have realized a very rapid capturing operation by mounting two X-Y actuators with different step sizes and by using an automatic system for washing the inside of the hollow punching needle while transferring the captured targets (cells or micro-dissections). The precisely controlled actuators (10 μm step, 3 μm accuracy) move a sample table (precise X-Y actuator). The rough but rapid actuators (100 μm step, 30 μm accuracy) deliver a micro-dissection captured by and held in a hollow punching needle into a reaction chamber. The actuators consist of an X-actuator to move the hollow punching needle mounted on an accurate Z actuator (10 μm step, 3 μm accuracy) and a Y-actuator (100 μm step, 30 μm accuracy) to move a 96-well plate containing the reaction chambers. A sample table is placed just above a base plate under which the video camera is attached to observe the sample. The hollow punching needle is made of a stainless steel tube (i.d.:110 μm, o.d.:460 μm) as shown in Fig. 1b, which attaches to an injector containing a buffer solution. A solenoid shutter controls the buffer flow from the injector to the needle. By pushing the plunger, a buffer flows into the hollow punching needle to eject a captured micro-dissection together with a buffer solution of 5 μL into a reaction chamber. The buffer flowing through the needle automatically washes its inner surface, keeping the needle clean and reducing the operating cycle time.

To test the system, we placed a tissue slice or cells on a scaffold sheet (polymer sheet) in a petri dish. Here we used collagen-coated polydimethylsiloxane (PDMS; 20 μm thickness) as the sheet (Fig. 1c). The edge of the hollow punching needle is manually adjusted to a position just below the upper surface of the scaffold sheet prior to a series of punching operations. This enables the effective punching of tissue without applying damage to the needle by the crush against the dish surface.

In the punching operation, after placing a target sample (tissue or cells) on the scaffold sheet, we push a calibration button. This calibrates the zero positions of the X-Y axis of the sample table and the Z-axis of the hollow punching needle. An image of tissue obtained by the video system is observed on the PC to define the capturing points with a cursor. Our system has three punching modes. The first is the manual mode (1): a tissue is moved on the sampling table to the position where the capturing point is beneath the hollow punching needle while observing the tissue image. The sampling bottom for capturing a micro-dissection is then pressed. The second is the random access mode (2); the selection of multiple capturing points in a target tissue is carried out using the cursor on a PC. The sampling bottom for punching them is then pressed to automatically recover the micro-dissections into the reaction chambers of a 96-well plate. The third is the automated matrix mode (3); the automatic capturing of micro-dissections from orderly arrayed points in a tissue is carried out by determining the starting point as well as the X and Y punching intervals of the matrix. We determine the number of capturing points along the X and Y axes prior to initiating punching. The maximum number of capturing points is currently 48, although this will increase to 384 in the near future. One sampling cycle includes the following motions: punching a tissue, moving the hollow punching needle to a reaction chamber in a titer plate, ejecting a micro-dissection together with buffer solution into a reaction chamber, and returning the needle to the original position. In the latter two automated modes, this process takes less than 5 s for one sampling cycle. The whole process for punching 48 points in a tissue can be carried out within 4 min. In addition, we verified the success rate of capturing micro-dissections by punching with this system. After PDMS coated on the dish was punched out, the presence of a PDMS piece punched out was confirmed with a microscope. In the five independent experiments, the capturing was succeeded in 202 trials out of 208. The success rate was 97.1%.

We checked the accuracy of the punched positions in the automated matrix mode by setting the capturing points to be arrayed at an interval of 300 μm along with the X and Y-axis, respectively. The holes produced in a tissue by the punching were checked to estimate the spatial accuracy of the punching operation. The center-to-center distances of the punched neighbor holes were 300 ± 4 μm for the X-axis and 298 ± 3 μm for the Y-axis. In the random access mode, the difference between the expected and the punched positions was within 10 μm.

As the hollow punching needle is made of stainless steel and is very tough compared to a glass capillary needle, it can repeatedly be used even for a plant tissue having hard cell walls. In addition, the inner diameter can be reduced to < 50 μm if necessary. We note that as a series of punching operations is carried out continuously with a single hollow punching needle, cross contamination among captured micro-dissections may occur by carrying over a part of a captured micro-dissection into the next sampling.

For the evaluation, we obtained a single dissection by punching cells. This was recovered in a reaction chamber via the washing system with a buffer solution. Then, a reference sample was obtained without punching cells but including the post-sampling buffer solution recovery process. We compared the gene expression levels for these two samples. Although the gene expression levels of the housekeeping genes for the second sample were not zero owing to the carry-over contamination, they were less than 1% of those of the first sample (Supplementary Table 1). We, therefore, concluded that the cross contamination was negligible.

### Gene expression profiles for adhered cells captured by punching and by pipetting after their removal from surfaces with reagent treatment

In studies of gene expression profiles, the cell or tissue sampling process frequently affects the results. It is straightforward to capture suspension cells such as blood cells for carrying out single cell analyses. However, most other cultured cells usually adhere to a dish surface. It is, therefore, necessary to remove adhered cells from the surface by reagents or mechanical scratching prior to gene expression analysis. Notably, this removal process may change their gene expression profiles. Our capturing system by punching is useful to pick up cells or micro-dissections of tissue in their native state by punching them together with a part of the scaffold sheet. We investigated the effects of sampling processes by punching, scratching, and reagent treatment methods on gene expression profiles for adhered HCT116 cells. For this, we cultured HCT116 cells in a dish covered with a collagen coated PDMS sheet.

**Figure 2:**
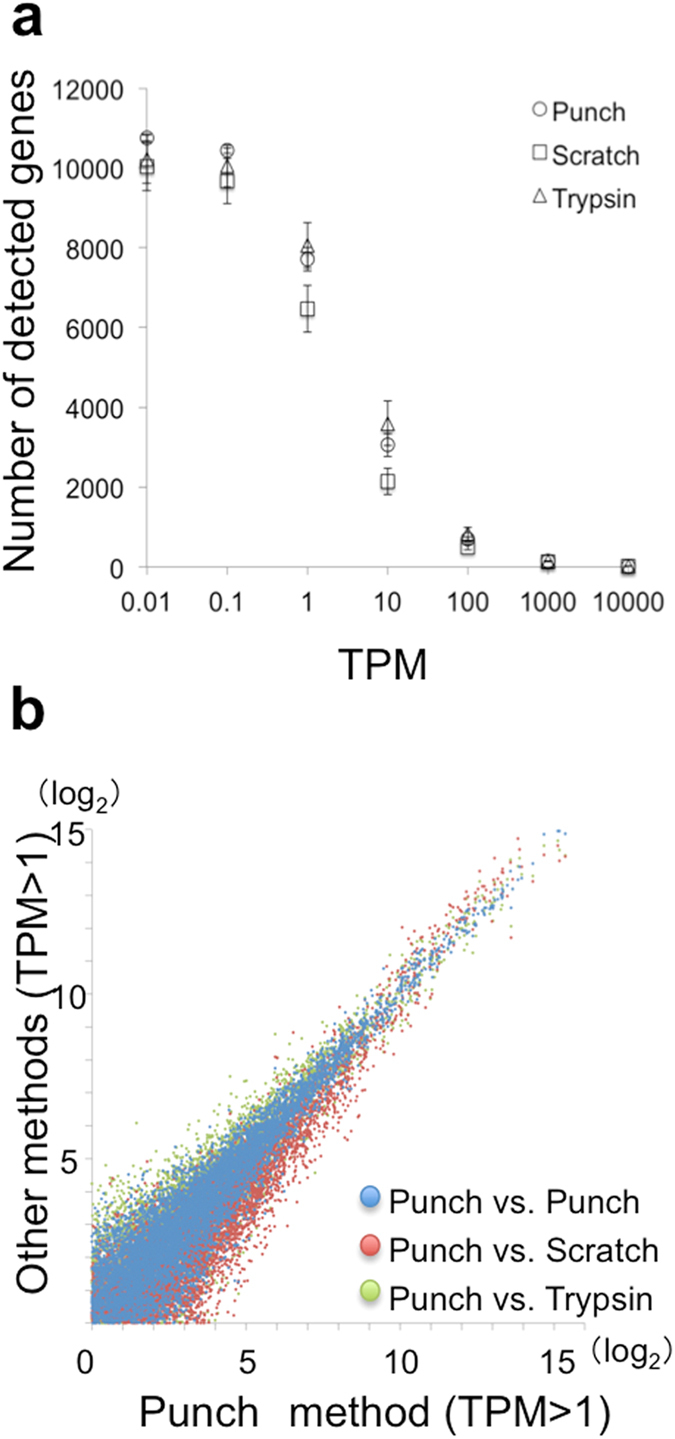
Number of genes identified and scatter plots for the three methods. (**a**) Numbers of observed gene species vs. minimum TPM. Error bars represent standard deviations. (**b**) Scatter plots of gene expression levels obtained with the punching method and the other methods (in Log2 TPM scale).

The punching and reagent treatment methods yielded similar numbers of detected gene species among those with expression levels over 1 transcript per million (TPM) (Fig. 2a) (punching: 7726 ± 296, trypsin treatment: 8069 ± 562) whereas the scratching method gave a much smaller number (6490 ± 582). Scatter plots of gene expression levels obtained by the various methods are shown in Fig. 2b. The Pearson’s correlation factors were 0.958–0.966 for samples obtained with the punching method, 0.913–0.953 for those obtained with punching and scratching, and 0.932–0.949 for samples obtained with punching and trypsin treatment methods.

The further precise evaluation was carried out by comparing the average gene expression levels as well as coefficients of variance (C.V.) for samples prepared with the three different methods. We calculated the ratios of the averaged gene expression levels obtained with the punching method to those produced by trypsin treatment. The ratios should shift from unity if they are dependent on the methods. Gene species were selected that exhibited ratios over 3 or less than 1/3. Notably, 74 genes met the criterion as listed in Table 1.

**Table 1:** Sampling method dependence of gene expression.

	Gene	Punch	Scratch	Trypsin
Group I	*HSPA8*	2109.4 ± 88.4	1481.8 ± 225.8	1088.9 ± 134.4
	*MINOS1*	153.2 ± 27.5	55.8 ± 9.0	81.1 ± 2.2
	AL109927.1	27.6 ± 4.2	5.2 ± 5.9	0
Group II	*OAZ1*	737.4 ± 87.8	885.2 ± 126.1	1443.1 ± 165.8
	*PPIB*	155.9 ± 27.4	234.4 ± 85.4	579.6 ± 68.2
	*H3F3A*	235.6 ± 41.8	236.9 ± 39.1	472.0 ± 57.6

The gene expression levels (TPM) for two groups obtained with the three different sampling methods are listed. Genes in groups I and II were expressed at a higher and lower level, respectively, in samples from the punching method than those obtained using the trypsin treated method.

**Besides above**, Group I included *C11orf98, BAG6, AATF, LIN7B, EVL, VPS13D, ARHGAP40, PRDM11* and *NEURL1B*.

Group II included *YBX1, PRR13, IMPDH2 PEBP1, XRCC5, CLPP, TAGLN2, FIS1, C19orf48, SEPT7, ARL16, TPM4, HNRNPK, SMARCE1, MALL, TRAM1, DARS, RP11-49K24.6, GPN3, PDIA3, TMED9, SRSF5, C1orf174, ABCE1, USE1, CCDC137, GPR89A, BRD2, RBBP8, TBC1D15, RBM10, ZNF576, WRB, FAM45A, EPS8, WDR76, WDR3, CD40, INSIG1, FES, EMD, SLC30A9, TROVE2, SMARCC1, ERP44, PCGF2, AUH, TMCO3, SCG2, BUD13, SCFD2, YAF2, RP11-544M22.13, TCAIM, DOK1, CXorf38, MFGE8, WAS* and *EFNB2*.

### Site-specific gene expression analysis of mouse brain tissue

**Figure 3:**
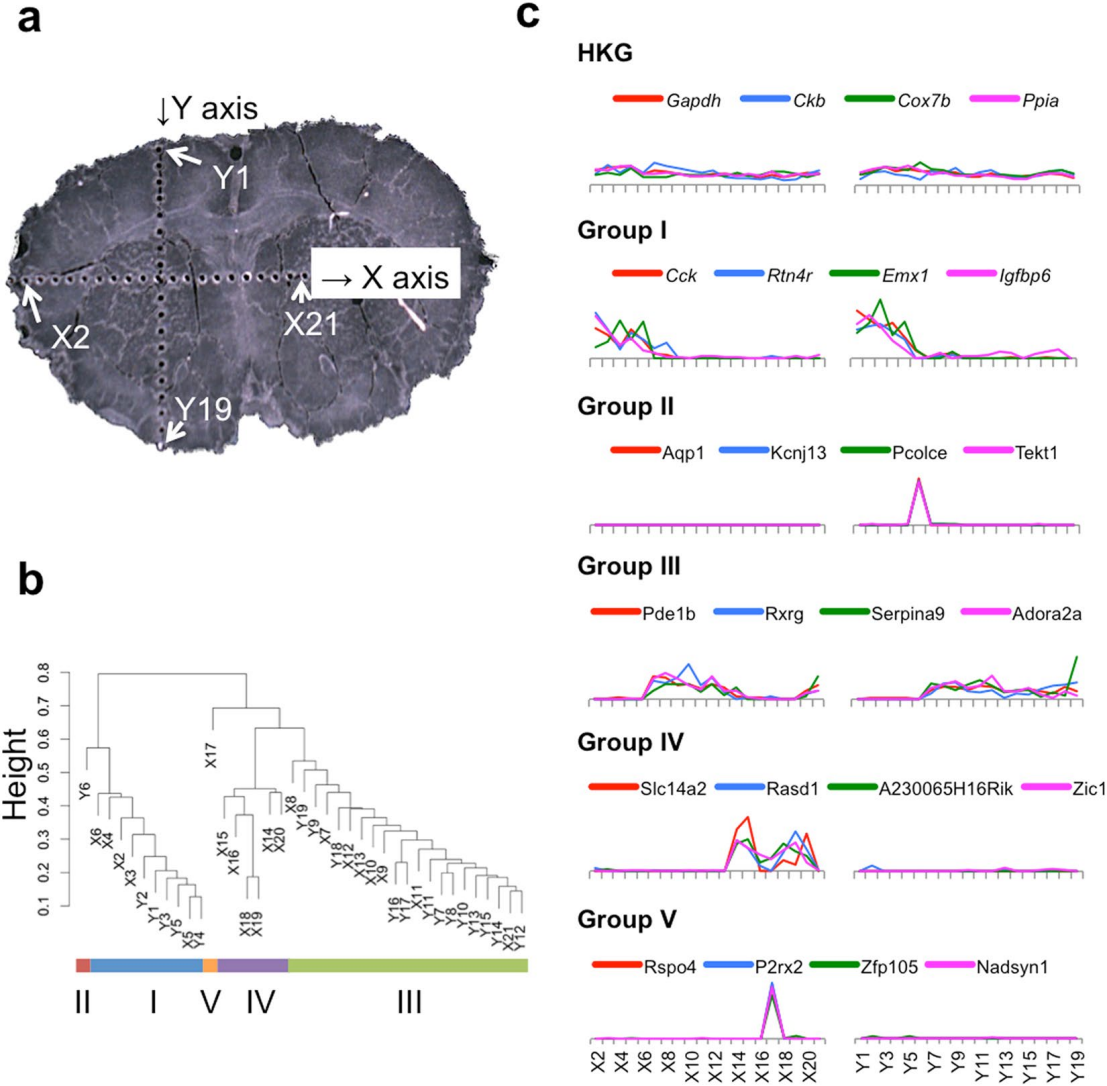
Site-specific gene expression. (**a**) Frozen mouse brain slice after the removal of micro-dissections. (**b**) Hierarchal clustering of micro-dissections based on gene expression. (**c**) Typical site-specific gene expression patterns. The expression levels were normalized by the average TPM for each gene.

For demonstrating the usefulness of the punching method for site-specific gene expression analyses, we produced numerous micro-dissections along two lines crossing a sliced frozen tissue of a mouse brain (20 μm thickness, Fig. 3a), which represents an adequate sample for site-specific analysis because of its anatomically complex characteristics. The obtained micro-dissections were from areas including the caudate putamen, cerebral cortex, corpus callosum, and medial septal nucleus. The cDNA libraries from the dissections were prepared and analyzed by RNA-seq (although the cDNA library from micro-dissection X1 could not be constructed). At first, we performed clustering of the micro-dissections to identify site-specifically expressed genes. We could classify the micro-dissections into five groups according to the similarities of their gene expression patterns as shown in Fig. 3b. Group I included micro-dissections X2-X6 and Y1-Y5. Group II included micro-dissection Y6. Group III included micro-dissections X14-X16 and X18-X20. Group IV included micro-dissections X7-X13, X21, and Y7-Y19. Group V included micro-dissection X17. The relative gene expression levels of site-specific genes at various points (normalized by the gene expression levels averaged over all micro-dissections) are displayed together with non-site specific genes (housekeeping genes, HKGs) in Fig. 3c. HKGs were stably expressed in all micro-dissections. The genes expressed in the micro-dissections belonging to group I included *Cck*, *Rtn4r*, *Emx1*, and *Igfbp6*. The genes *Aqp1*, *Kcnj13*, *Pcolce*, and *Tekt1* were expressed only in micro-dissection Y6 (group II). The genes *Pde1b*, *Rxrg*, *Serpina9*, and *Adora2a* were specific to group III. The genes specific to group IV were *Slc14a2*, *Rasd1*, *A230065H16Rik*, and *Zic1*. Genes including *Rspo4*, *P2rx2*, *Zfp105*, and *Nadsyn1* were specific to group V being expressed only in micro-dissection X17.

## Discussion

The presented capturing system realized the accurate and rapid sampling of micro-dissections from a tissue. The key was the use of two independent X-Y actuator systems with different moving steps, as well as an automated washing system with a buffer flow for cleaning a hollow punching needle. The automated washing enabled the repeated use of the needle more than 1,000 times by using a scaffold sheet to avoid its being crushed against a dish surface. Currently, the maximum number of micro-dissections captured in one continuous operation is limited to 48, which can be increased to 384 or more by changing the operating software. It takes 5 s for one sampling cycle, which is very rapid compared to the time reported for other sampling methods. It will likely be possible to reduce the time to less than 3 s by optimizing the actuators.

Although LCM is a powerful method to produce micro-dissections from a tissue slice, it has the following severe limitations. (1) It can be used only for a tissue slice thinner than 200 μm. (2) The target tissue slice should be fixed on a slide with ethanol or formalin. (3) It is very time consuming because it takes 5 min to capture one micro-dissection^23^. These drawbacks can be overcome with our punching method. It applies to any types of samples, including fresh, frozen, and fixed samples. It takes only 5 sec to capture one micro-dissection, which is very powerful for site-specific analyses because many micro-dissections have to be obtained for a site-specific gene expression analysis. For example, it takes only 8 minutes to capture 100 micro-dissections with the punching method, while LCM takes more than 8 hours for that. A long capturing time is undesirable because of the probable degradation of the sample in the capturing process^19^. Our method fit to the rapid capturing of micro-dissections from any types of tissue samples.

Presently, the punching points are determined by observing a target with a video camera, which is not suitable for obtaining a fluorescence image. We are planning to improve the system for realizing the capturing operation with fluorescence image information obtained using a fluorescence microscope before sampling. The most time-consuming process lies in determining the capture points. Therefore, the planned improvement is expected to reduce the sampling time significantly. From the viewpoint of a massively parallel analysis, the matrix sampling mode appears better than the random access sampling mode because it does not require time for determining the capture points.

The standard method for removing adhered cells from a surface usually relies upon trypsin digestion. However, there have been few reports regarding the effects of the reagent treatment on gene expression profiles. The punching method does not administer any damage to a target during sampling because the target is captured together with the scaffold sheet it is adhered on. We confirmed that the majority of gene expression profiles are similar between the samples prepared by reagent treatment and punching. However, we still identified many genes that changed their gene expression levels based on the removal processes as shown in Table 1. For example, the gene *PPIB* increased by three-fold from 155.9 ± 27.4 (punching) to 579.6 ± 68.2 (trypsin treatment). Peptidylpropyl isomerase B (*PPIB*) encoding cyclophilin B protein is known as a housekeeping gene stably expressed in adhesion cells^24^. Our result suggested that the gene expression of *PPIB* changed its expression level depending on the capturing method. Trypsin treatment has several processes, including incubation, centrifugation, and suspension. It takes at least 30–60 min from the addition of trypsin to the lysis of captured cells, while it can be done in 5 sec with our punching method. The increase of the *PPIB* expression level by the trypsin method might reflect the stress in the sample preparation processes. *PPIB* plays an important role in the collagen synthesis. Therefore, it suggested that the increase of the expression level was due to synthesize the collagen, which was damaged by trypsin treatment. Furthermore, although scratching was thought to be a rough sampling method compared to reagent treatment, the difference produced between reagent treatment and punching was occasionally much greater than that produced by the scratching and punching methods. Therefore, sampling by the punching method is recommended to obtain adhered cells in natural conditions.

In the experiments analyzing single cells or micro-dissections punched from real samples, it is impossible to prepare the same samples for the analysis. Therefore, in the site-specific gene expression analysis, it is necessary to distinguish the real changes of gene expression levels due to the site specificity from non-site specific changes. We set two criteria for the site-specific gene expression changes. They are; 1) the changes are much bigger than those observed in uniform samples, 2) at least there are two or more gene species change in a similar manner. Although the coefficient of variation is frequently used as an indicator of the uniformity for gene expressions, we used the ratios of maximum expression levels to the average expression levels (Max/Ave) because they are very sensitive for the site-specific changes. The Max/Ave values for various genes are plotted in Supplementary Fig. S1 for three different samples (total RNA (500 pg), punched cultured cells and punched mouse brain slice). We used total RNA of HCT116 as a model sample because one micro-dissection of brain slice contains about 30 cells which include approximately 500 pg of total RNA. The plots obtained for total RNA and cultured cells are quite different from that for mouse brain slice. The several genes in mouse brain sample showed much bigger Max/Ave ratios compared to other samples. We considered these genes would be good indicators for discussing the site specificities. They were classified into several groups according to anatomical brain regions. Their gene expression levels changed from site to site while house keeping genes stayed almost constant over the whole region. Because these results were coincided with the test for differential expression using DESeq2, we perform the test for differential expression between the sampling sites and assesed the site-specific genes in the mouse brain.

**Figure 4:**
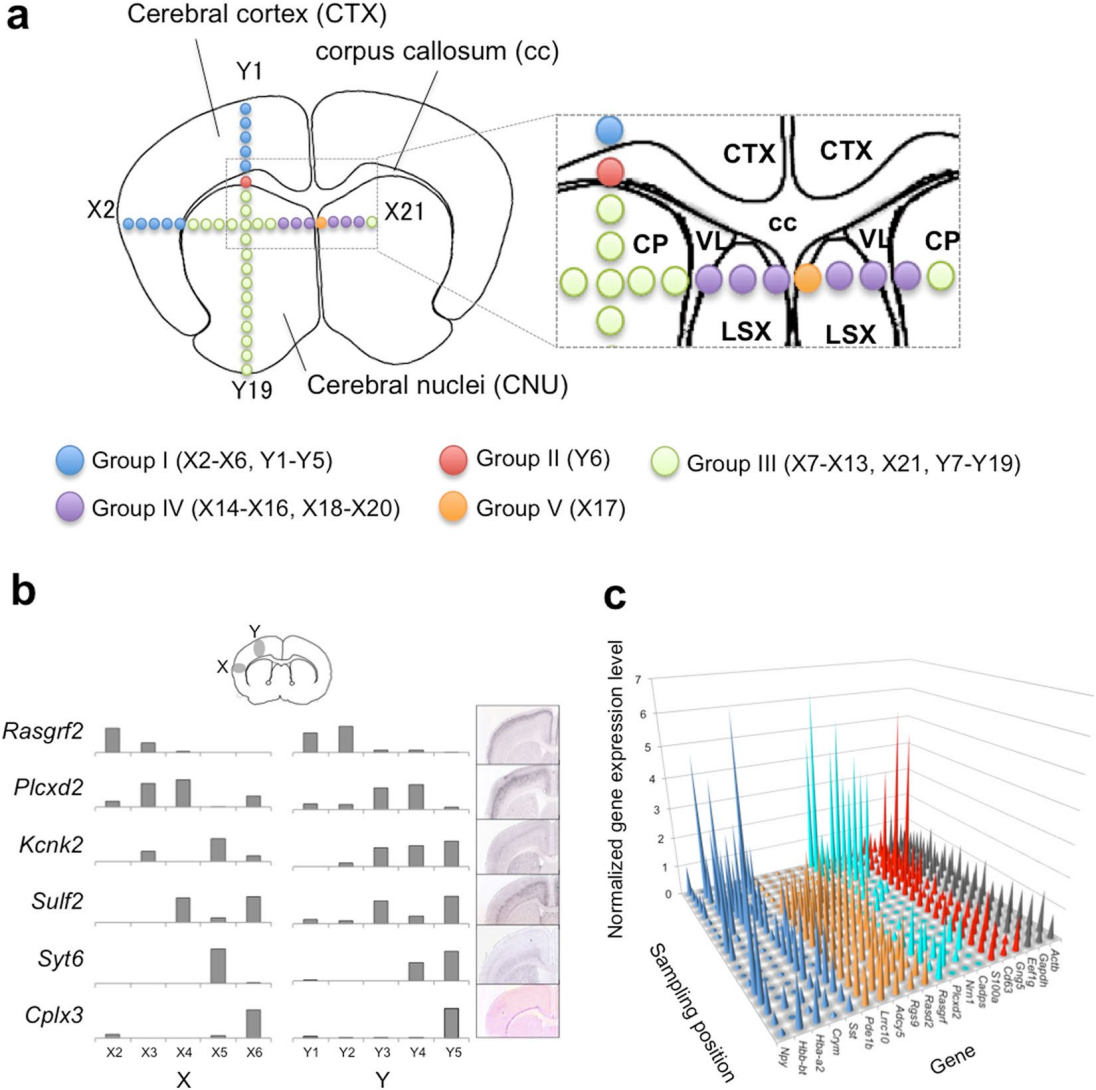
Anatomical regions and their gene expression. (**a**) Sampling points and brain anatomical regions. (**b**) Layer-specific genes expressed in the selected 10 micro-dissections from the cerebral cortex together with *in situ* hybridization images from the Allen Institute (the Allen Mouse Brain Atlas; http://mouse.brain-map.org/). (**c**) The landscape for site-specific gene expression. (x axis; gene species, y axis; position, z axis; gene expression levels normalized by the average TPM of each gene). *Npy*, *Hbb-bt*, *Hba-a2*, *Crym*, and *Sst* expression changed dynamically from site to site (blue). *Pde1b*, *Lrrc10b*, *Adcy5*, *Rgs9*, and *Rasd2* expressed in Y7-Y19 (orange, group III). *Rasgrf2*, *Plcxd2*, *Nrn1*, and *Cadps2* expressed in Y1-Y5 (cyan, group I). *S100a1*, *Cd63*, and *Gng5* expressed in Y6 (red, group II). Housekeeping genes such as *Eef1g*, *Gapdh*, and *Actb* expressed stably in all micro-dissections (black).

The brain tissue slice utilized in this study contained the following three regions: cerebral cortex (CTX), corpus callosum (cc), and cerebral nuclei (CNU). CNU consists of three regions (caudoputamen; CP, ventriculus lateralis; VL, and lateral septal complex; LSX) as shown in Fig. 4a. The positions from which the micro-dissections were obtained are also shown in the figure. We clustered the micro-dissections into five groups (group I ~ group V) by gene expression patterns, which coincided with the anatomical regions (Fig. 4a). Group I including the X2-X6 and Y1-Y5 micro-dissections corresponded to CTX. Marker genes for CTX such as *Cck* and *Nm1* were expressed strongly in this group. Group II included only one micro-dissection, Y6. This is because cc was a narrow region judging from a tissue image. Although we could not identify marker genes for cc from the Allen Brain Atlas^25^, many site-specific genes were observed as listed in Supplementary Table 2. They include genes such as *Ttr* and *Aqp1* that are well known as marker genes of the choroid plexus, which is located on the border between cc and VL. VL is a region filled with cerebrospinal fluid that is produced in the choroid plexus. Therefore, micro-dissection Y6 must include cells belonging to not only cc but also the choroid plexus. Genes expressed in groups III, IV, and V were from CNU. CNU includes the two regions of CP and LSX. Marker genes for CP, such as *Cd4* and *Adra2a*, were expressed in group III. Marker genes for LSX, such as *Prkcd*, were expressed in groups IV and V.

CTX contains several superficial layers. We investigated whether we could recognize these superficial layers by the punching method. We carefully checked the gene expression profiles obtained from micro-dissections X2-X6 and Y1-Y5, which might be from CTX (Fig. 4b). According to a published report^26^, the superficial layers are classified to six layers from layer I to layer VI (VIa, VIb). It was also reported that genes such as *Rasgrf2*, *Plcxd2*, *Kcnk2*, *Sulf2*, and *Syt6* represented marker genes for specifying the layers. These gene expression levels are displayed by various methods in Fig. 4b. According to the reference, *Rasgrf2* expresses strongly in superficial layers II/III. Therefore, these layers must be in X2, Y1, and Y2. As *Plcxd2* expresses in superficial layers IV, V, and VI, micro-dissections X3, X4, Y3, and Y4 likely arise from those layers. As *Kcnk2* expresses strongly in superficial layer Va, micro-dissections X5, Y3, Y4, and Y5 probably derive there from. *Sulf2* expressed in superficial layers V and VIb, which appeared in micro-dissections X4, X6, Y3, and Y5. *Syt6* (a marker gene of superficial layer VI) expressed in micro-dissections X5, Y4, and Y5 (superficial layer VI) and *Cplx3* (a marker gene of superficial layer VIb) expressed in X6 and Y5. As the size of the micro-dissections was rather large (0.1 mm in diameter) and the sampling positions were not optimized for observing the layers, a single micro-dissection might include cells from several layers. It would be possible to obtain much better site-specific data for layer analysis by selecting the sampling positions coupled with imaging technology. The use of a narrower punching needle may also be helpful for analyzing the detailed structure of tissue for obtaining gene expression data together with spatial information.

We also noted that some genes, such as *Sst*, *Npy*, and *Hbb*-*bt* were markedly changed according to sampling positions. These changed substantively from position to position in the same anatomical region and even when differing by only 300 μm from the neighboring position (Fig. 4c). As gene expression occasionally changes in a living tissue and the obtained result represents a snapshot of the gene expression, the drastic changes might be due to time fluctuation of the gene expression. More data will be required to confirm the reason for the expression change phenomenon.

We obtained many site-specific gene expression patterns with various slices. To show that the site-specific change discussed above is not a special case, we showed another site-specific gene expression pattern from mouse olfactory bulbs in Supplementary Fig. S2.

Notably, as it is possible to extract proteins together with transcripts from micro-dissections, it will likely be possible to simultaneously obtain the spatial information of transcripts as well as proteins for a given tissue using the capturing system. Currently, the availability of precise spatial transcriptomics data might provide new insight into tissue gene expression and function. Our final goal is to construct a three-dimensional transcriptome as well as a protein map of tissue with a high spatial resolution, and we expect that the punch sampling system will play an important role in this endeavor.

## Methods

### Hollow punching needle and related materials

The hollow punching needle was made of stainless steel purchased from Castec (Kanagawa, Japan). It had a knife-edge of 5 μm diameter or less to cut all biomaterials smoothly. It was connected to an injector filled with a buffer solution by a polytetrafluoroethylene tube from Nichias (Tokyo, Japan). The whole punching system was designed by our team and produced by Sanyu (Ibaragi, Japan). Before capturing cells or micro-dissections of tissue, we washed the inside of the hollow punching needle with 70% EtOH, RNase Zap (Thermo Fisher Scientific, MA, USA), and PBS (Gibco, NY, USA). The sample table was equipped with a dish, the diameter of which was approximately 35 mm. We coated its inner surface with a PDMS film of 20–100 μm thickness. Occasionally, double layers of PDMS film were generated to capture a micro-dissection together with a part of the film.

The amount of PDMS (Sylgard 184: Dow Corning Corp., MI, USA) used was 100 μg, which provided a thickness in the range from 20–100 μm. PDMS and its cross-linker were mixed thoroughly at a ratio of 10:1 (w/w) and then degassed. The PDMS mixture (100 μL) was placed in the center of a polystyrene dish (AGC Techno Glass, Iwaki, Japan; 35 mm in diameter) to be spin-coated at 500 rpm for 10 s and at 3,000 rpm for 15 s with a SpinCoater (Mikasa, Tokyo, Japan). The dish coated with PDMS film was kept at 70 °C for at least 2 h. Before culturing cells, the coated dish was washed with 70% EtOH and then coated with Cellmatrix type IV (Nitta Gelatin Inc., Osaka, Japan) including collagen according to the manufacturer’s protocol.

### Cell culture and removal from a dish

We cultured the HCT116 cell line in a dish coated with PDMS film. The medium included DMEM (Gibco, NY, USA) supplemented with 10% (v/v) fetal calf serum (Gibco) and 1% L-glutamine (Gibco). The cells were kept at 37 °C in a humidified atmosphere including 5% CO_2_ for 30 h. The cell concentration in the dish was approximately 10^5^ cells per mL after cultivation. The cultivated cells were washed twice with 2 mL PBS to keep them on the coated material. The wash buffer was removed and 2 mL of fresh PBS was added to keep the cells hydrated.

The dish containing the cultured cells was set on a sample table in the capturing system. After capturing cells by punching, they were transferred into reaction chambers in a 96-well plate filled with 3 μL RT-PCR grade water (Ambion, CA, USA). Then the plate was centrifuged for 10 s at 2,000 rpm with PlateSpin II (Kubota, Tokyo, Japan).

In the trypsin treatment method, cells were incubated with 0.05% trypsin (Gibco) for 5 min at 37 °C. Then, the cells were resuspended in PBS to be picked up with a micropipette under a microscope according to Huang’s method^9^. The collected cells were placed in reaction chambers filled with 3 μL RT-PCR grade water.

### Preparation of sliced frozen tissues from mouse brain

All mice (ICR, male, > 2 months old, Tokyo Laboratory Animals Science Co.,Ltd.,Tokyo, Japan) were treated according to the protocols approved by the Committee for Animal Experimentation of the School of Science and Engineering at Waseda University (No. 2016-A124) and in accordance with the law (No. 105) passed by and notification (No. 6) of the Japanese Government. Thus, these studies were approved by the School of Science and Engineering at Waseda University.

The mice were euthanized and brains were immediately isolated. The mouse brains were embedded in SCEM solution (Leica Microsystems, Tokyo, Japan), rapidly frozen in liquid nitrogen, and stored at −80 °C prior to cryosectioning. The embedded surface was trimmed with a cryo-microtome (Leica) then covered with a Cryo-film (SECTION-LAB, Hiroshima, Japan) and cryosectioned at a thickness of 20 μm. The sliced brain tissue covered with a Cryo-film was laid on a polystyrene dish placed on the sample table. Micro-dissections produced at a 300-μm pitch were recovered together with the Cryo-film in the 96 reaction chambers filled with 3 μL of RT-PCR grade water. The 96-well plate was centrifuged for 10 s at 2,000 rpm with PlateSpin II to immerse the micro-dissections in water. All the processes, from the production of a frozen slice of brain tissue to capturing 39 micro-dissections of the tissue and recovering them in the reaction chambers, were carried out within 10 min.

### Quantitative analysis of cDNA by qPCR to determine the degree of carry-over contamination

As a reference, we analyzed cDNAs for a housekeeping gene (*EEF1G*) quantitatively by qPCR with the Step One Plus real-time PCR system (Applied Biosystems, CA, USA). qPCR was carried out with a 10-μL solution containing 1 × PrimeTime Gene Expression Master Mix (Integrated DNA Technologies (IDT, IA, USA) and PrimeTime qPCR Assays (IDT). The reaction mixture was heated at 95 °C for 3 min, followed by 40 thermal cycles of 95 °C for 5 s and 60 °C for 30 s. Reference DNA samples (IDT) were analyzed together with the samples for calibrating the qPCR results. The sequence of the reference DNA for *EEf1G* is: (5′-CTGTG GTACT CAGAG TATCG CTTCC CTGAA GAACT CACTC AGACC TTCAT GAGCT GCAAT CTCAT CACTG GAATG TTCCA GCGAC TGGAC AAGCT GAGGA AGAAT GCCTT CGCCA GTGTC ATCCT TTTTG GAACC AACAA TAGCA GCTCC ATTTC TGGAG TCTGG GTCTT CCGAG GCCAG GAGCT TGCCT TTCCG CTGAG TCCAG ATTGG CAGGT GGACT ACGAG TCATA CACAT GGCGG AAACT GGATC CTGGC AGCGA GGAGA CCCAG ACGCT GGTTC GAGAG TACTT TTCCT GGGAG GGGGC CTTCC AGCAT GTGGG CAAAG CCTTC AATCA GGGCA AGATC TTCAA GTGAA CATCT CTTGC CATCA CCTAG CTGCC TGCAC CTGCC CTTCA GGGAG ATGGG GGTCA TTAAA GGAAA CTGAA-3′). Primer sets (EEF1G Hs.PT.58.2818602.gs) were purchased from IDT.

### cDNA library construction and sequencing by the Bead-seq method

We converted all mRNA in micro-dissections as well as cells into cDNA using the Bead-seq method^8^. All the processes from the production of cDNA libraries to their amplification and purification were carried out automatically using a Caliper Zephyr Compact Liquid Handler (Perkin Elmer, CA, USA) coupled with a homemade operation program based on the Bead-seq method. Ninety-six samples could be treated simultaneously with the automated system.

Sequencing libraries were constructed using the Nextera XT DNA Sample Preparation Kit (Illumina, CA, USA) to yield 75 bp paired-end cDNA sequence reads with Illumina MiSeq.

### Data analysis

We trimmed the adapter sequences in all the sequence reads using Cutadapt 1.9.1^27^. The trimmed sequence reads were aligned to the Ensembl human reference genome (GRCh38 ver.76) for cell line samples and the Ensembl mouse reference genome (GRCm38 ver.84) for mouse brain tissue samples including the *ERCC* sequences using bowtie2 2.2.3^28^ with the default parameters. The gene expression levels, given as TPM, were calculated using RSEM 1.2.28^29^ with a transcriptome reference obtained from Ensembl. We removed mitochondrial genes and ribosomal genes from the TPM data because they fluctuated from sample to sample and gave high abundances. We also removed genes with zero TPM in every micro-dissection.

For the gene expression analysis of HCT116, we used genes having TPM over 1. The averages, the coefficients of variation (C.V.), and the correlations among gene expression profiles were calculated.

For the gene expression analysis of mouse brain, we used genes exhibiting TPM over 50 in at least one micro-dissection. These included 6,176 genes. Each micro-dissection was assigned to a group based on Euclidean distance (ward.D2). Using DESeq2^30^, we identified 2,076 group-specific genes. The gene expression levels averaged over the micro-dissections in each group as well as their C.V. were calculated. The group-specific genes ranked at the top in DESeq2 results and exhibiting C.V. for each group over 1.5 were selected as the representatives of each group (Group1: *Cck*, Group2: *Ttr*, Group3: *Pde1b*, Group4: *Slc14a2*, Group5: *P2rx2*). The coefficients of correlation were then calculated for these genes (Supplementary Table 2).

Another easy way of selecting site-specific gene species is calculation of Max/Ave values of gene expression levels.

## Electronic supplementary material

Supplementary Information

Supplementary Table 1

Supplementary Table 2
