# Supplementary material for: Site-specific gene expression analysis using an automated tissue micro-dissection punching system
Source: Sci Rep. 2017 Jun 28;7:4325. doi: 10.1038/s41598-017-04616-6 (PMC5489509; doi:10.1038/s41598-017-04616-6)
Supplement: Supplementary file 1 — Supplementary Information [file 41598_2017_4616_MOESM1_ESM.doc]

Supplementary Information

**Site-specific gene expression analysis using an automated tissue micro-dissection punching system**

Takuya Yoda^1^, Masahito Hosokawa^2,3^, Kiyofumi Takahashi^2^, Chikako Sakanashi^2^, Haruko Takeyama^1,2,4^ & Hideki Kambara^2^, *

^1^ Department of Life Science and Medical Bioscience, Waseda University, 2-2 Wakamatsu-cho, Shinjuku-ku, Tokyo 162-8480, Japan.

^2^Research Organization for Nano &Life Innovation, Waseda University, 513 Waseda-tsurumaki-cho, Shinjuku-ku, Tokyo, 162-0041, Japan.

^3^ PRESTO, Japan Science and Technology Agency (JST), 5-3 Yonban-cho, Chiyoda-ku, Tokyo 102–0075, Japan

^4^ Computational Bio Big-Data Open Innovation Laboratory, AIST-Waseda University, 3-4-1 Okubo, Shinjuku-ku, Tokyo 169–0072, Japan

*Correspondence and requests for materials should be addressed to H.K. (email:h.kanbara@kurenai.waseda.jp).


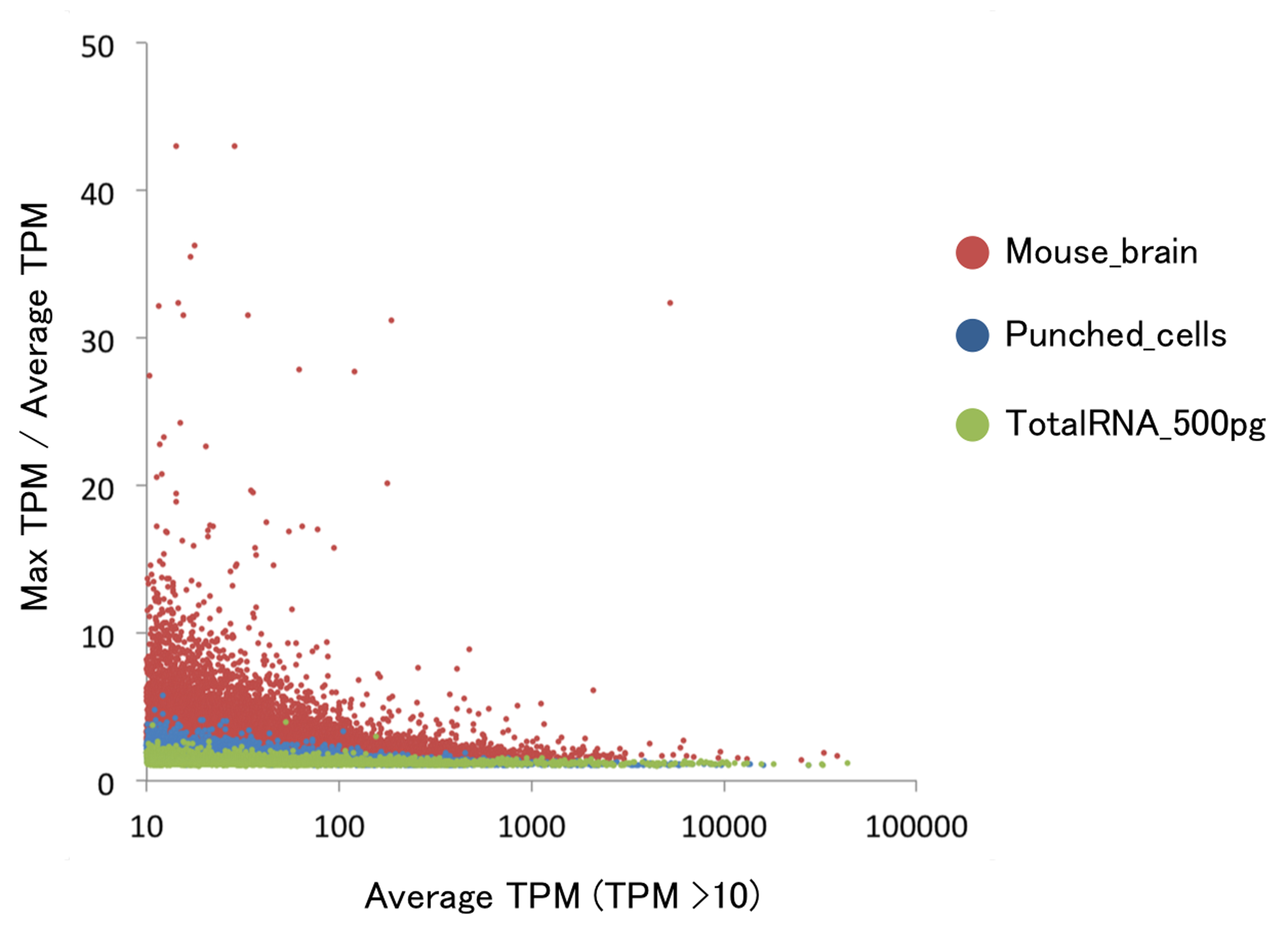


Supplementary Fig. S1 Scatter plots of correlations between average and Max / Average values of TPM.

Each value was estimated from RNA-seq data obtained from 500 pg total RNA (HCT116), the cells directly punched from a culture dish (HCT116), and the brain dissections punched from a frozen mouse brain slice by the capturing system.


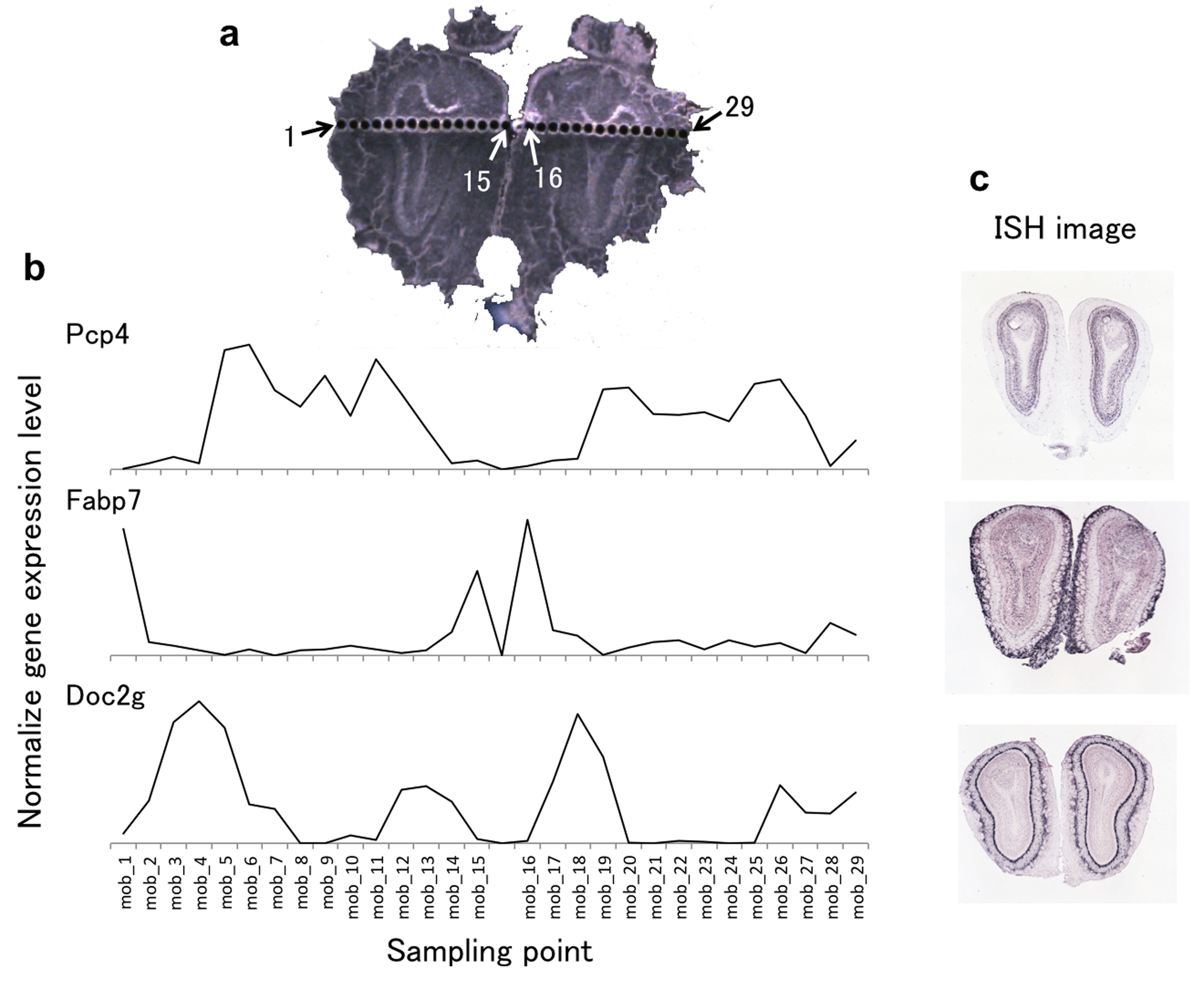


Supplementary Fig. S2 Site-specific gene expression profiles in the 29 micro-dissections punched from mouse olfactory bulbs.

(a) The microphotograph image of mouse olfactory bulbs. The number indicates the sampling number of micro-dissections.

(b) The line profiles of site-specific expression profiles of olfactory bulb marker genes. The expression levels were normalized by the average TPM for each gene.

(c) *In situ* hybridization images for each marker gene. These images were obtained from the Allen institute (the Allen Mouse Brain Atlas; <http://mouse.brain-map.org/>)(1).

**Reference**

1. Lein, E.S., et al. Genome-wide atlas of gene expression in the adult mouse brain. *Nature* **445**, 168-176 (2007).
